# Development and Validation of a Multi-Algorithm Analytic Platform to Detect Off-Target Mechanical Ventilation

**DOI:** 10.1038/s41598-017-15052-x

**Published:** 2017-11-03

**Authors:** Jason Y. Adams, Monica K. Lieng, Brooks T. Kuhn, Greg B. Rehm, Edward C. Guo, Sandra L. Taylor, Jean-Pierre Delplanque, Nicholas R. Anderson

**Affiliations:** 1 Division of Pulmonary, Critical Care, and Sleep Medicine, University of California Davis, Sacramento, CA USA; 2 School of Medicine, University of California Davis, Sacramento, CA USA; 3 Department of Computer Science, University of California Davis, Davis, CA USA; 4 Department of Public Health Sciences, Division of Biostatistics, University of California Davis, Davis, CA USA; 5 Department of Mechanical and Aerospace Engineering, University of California Davis, Davis, CA USA; 6 Department of Public Health Sciences, Division of Informatics, University of California Davis, Davis, CA USA

## Abstract

Healthcare-specific analytic software is needed to process the large volumes of streaming physiologic waveform data increasingly available from life support devices such as mechanical ventilators. Detection of clinically relevant events from these data streams will advance understanding of critical illness, enable real-time clinical decision support, and improve both clinical outcomes and patient experience. We used mechanical ventilation waveform data (VWD) as a use case to address broader issues of data access and analysis including discrimination between true events and waveform artifacts. We developed an open source data acquisition platform to acquire VWD, and a modular, multi-algorithm analytic platform (ventMAP) to enable automated detection of off-target ventilation (OTV) delivery in critically-ill patients. We tested the hypothesis that use of artifact correction logic would improve the specificity of clinical event detection without compromising sensitivity. We showed that ventMAP could accurately detect harmful forms of OTV including excessive tidal volumes and common forms of patient-ventilator asynchrony, and that artifact correction significantly improved the specificity of event detection without decreasing sensitivity. Our multi-disciplinary approach has enabled automated analysis of high-volume streaming patient waveform data for clinical and translational research, and will advance the study and management of critically ill patients requiring mechanical ventilation.

## Introduction

Acute respiratory failure is the most common reason for intensive care unit (ICU) admission in the U.S. and is associated with an average in-hospital mortality of approximately 30% and $54 billion in attributable yearly costs^1,2^. Mechanical ventilation (MV) provides life-saving therapy but if delivered improperly can cause ventilator-induced lung injury (VILI)^3^, and substantial patient distress if patient effort and MV support are not well-matched (known as patient-ventilator asynchrony (PVA)), that may further promote lung injury^4,5^. One of the principle mechanisms of VILI is known as volutrauma, whereby delivery of excessive tidal volumes (TV, the volume delivered by the ventilator with each breath) results in pathologic alveolar distention, cellular injury, and the development of diffuse lung injury with many of the pathologic and clinical hallmarks of the acute respiratory distress syndrome (ARDS), a common and severe form of diffuse lung injury associated with mortality of up to 50%^6^. Excessive distention of lung tissue may result from inappropriately prescribed ventilator settings, excessive patient effort, or from subtypes of PVA that result in incomplete exhalation in between breaths, trapping gas in the lungs and further distending tissue^3–5,7^. Randomized controlled trials in patients with ARDS suggest that targeting a low tidal volume ventilation (LTVV) strategy of approximately 6 ml/kg of predicted body weight and controlling PVA improve survival although studies to date have not been able to separate the effects of excessive TV from those of PVA^8,9^. Studies in ventilated patients without ARDS suggest that a LTVV strategy reduces the development of respiratory complications and hospital-acquired ARDS^10–12^. Despite over 15 years of accumulating evidence, the expected widespread translation of lung-protective MV targets into clinical practice has not occurred^13–15^.

Despite its high prevalence, cost, and associated suffering, MV remains difficult to study and no well-validated, widely available analytic or clinical decision support tools exist to facilitate patient-specific, precision management of MV. Waveform data from MV- and most other life support devices- are not generally available in the electronic health record (EHR), limiting the ability to develop analytic tools. MV data from clinical studies have typically been hand-recorded only a few times per day, representing a gross under-sampling of patients who routinely take more than 20,000 breaths per day^8,9,14,16^, and most studies have been unable to collect and analyze the rich streams of ventilator waveform data (VWD) used by clinicians at the bedside to diagnose and manage pathologic patient-ventilator interactions. Manual analysis of large volumes of physiologic waveform data is limited by its labor-intensive nature, and recent data suggest that ICU clinicians perform poorly when asked to identify common forms of PVA through visual inspection of VWD, further supporting a need for standardized, automated analytic tools^17^.

A number of small studies have collected VWD using intrusive (e.g., laptop computers) or non-scalable methods of data acquisition, using a variety of analytic approaches to classifying PVA from manual annotation to power spectral analysis to the application of proprietary waveform analysis software^18–27^. These studies have demonstrated an important proof of concept, namely that MV waveform data are rich in historically unrecorded information pertinent to patient-ventilator interactions, and that analysis of PVA and other forms of “off target” ventilation (OTV) may reveal associations with important clinical outcomes and processes of care. Studies to date have been limited by lack of access to ventilator data, intrusive data collection methods that may introduce observer bias^28,29^ and limit the feasibility of continuous longitudinal data collection, limited clinical validation of algorithm performance, inability to distinguish between OTV subtypes, and lack of defined analytic mechanisms to distinguish between true OTV events and waveform artifacts that may result in false positive event classification^18–27^. As these issues are not unique to MV, the development of improved MV waveform analysis software serves as a generalizable use case for the challenges facing the broader development of healthcare “big data”-specific analytics and decision support systems including barriers to data access, transmission, standardization, security, storage, and computation; incorporation of clinician-informed knowledge and heuristics into algorithms able to transform complex, high-volume raw data into actionable information while minimizing false alarms; and the development of well-engineered software solutions that allow extensibility, integration with other systems, and ultimately, provisioning of clinical decision support to the point of care^30–32^.

In this study, we aimed to develop and validate an integrated MV waveform data acquisition and analysis platform capable of unobtrusive, continuous data collection and breath-by-breath classification of OTV to support clinical outcomes research, translational patient phenotyping^33^, continuous quality improvement, and precision medicine through clinical decision support. We assembled a multi-disciplinary team including clinicians, engineers, computer scientists, and informaticists, and developed an extensible, modular analytic engine, referred to as the ventilator multi-algorithm analytic platform (ventMAP), using rule-based logic derived from clinical bedside interpretation of MV waveforms^4,5^ to determine both inspiratory and expiratory TV, two well-recognized forms of PVA associated with hyper-inflation of the lungs, and several common types of VWD “clinical artifacts” that morphologically resemble true PVA.

**Figure 1:**
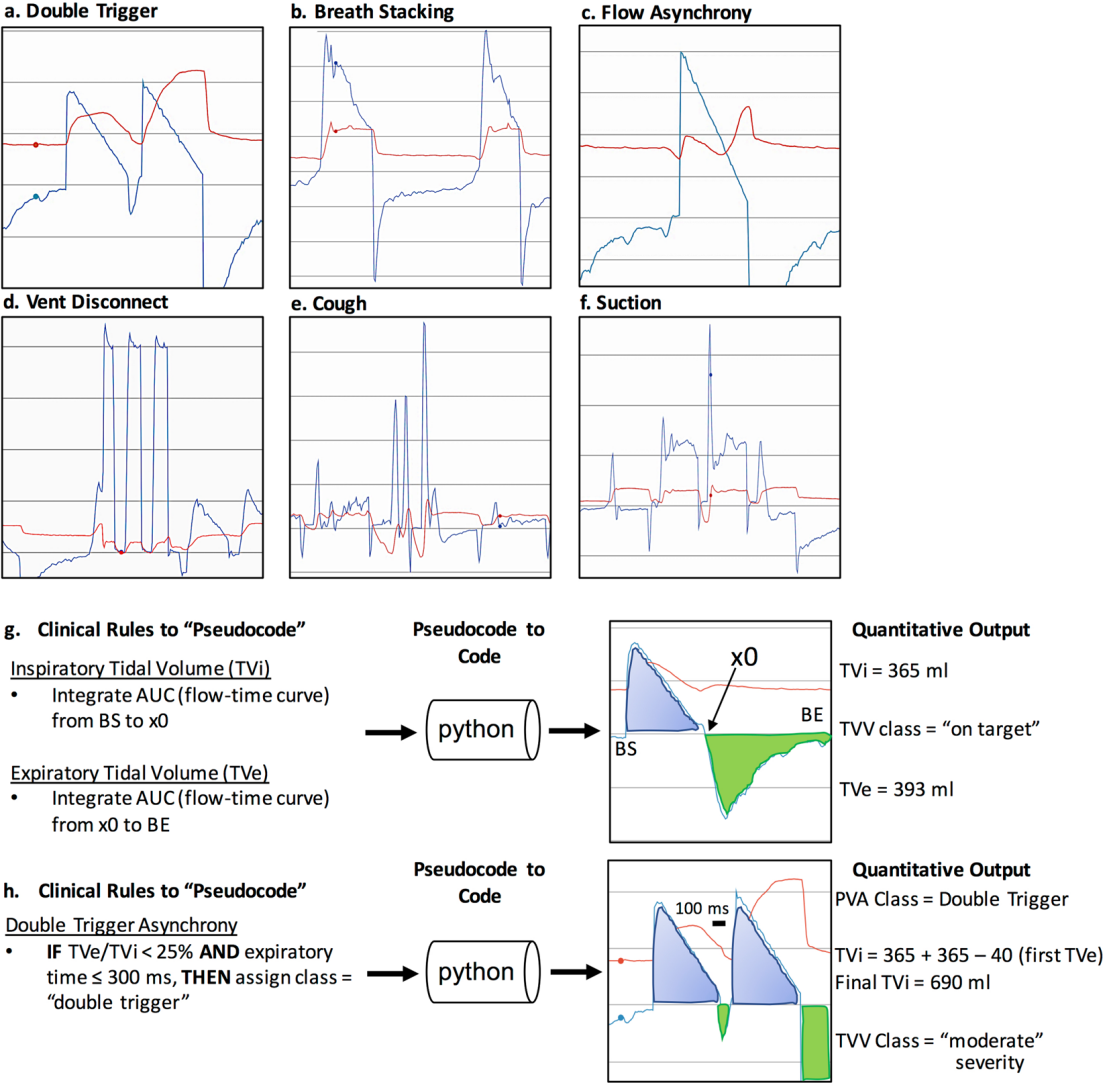
Examples of waveforms and algorithm development. (**a**–**f**) Common subtypes of off-target ventilation including patient-ventilator asynchronies and clinical “artifacts”. Vertical axis displays either pressure (red) or flow (blue) and horizontal axis displays time. (**g**,**h**) Example of ventMAP algorithm development workflow and rules engine output including tidal volumes and double trigger. x0, the point at which flow changes from inspiration to expiration; TV, tidal volume; TVV, tidal volume violation; TVi, inspiratory tidal volume; TVe, expiratory tidal volume; ms, milliseconds.

Our work has focused on the classification of events thought to contribute to VILI through excessive distention of lung tissue including excessive TV (referred to as tidal volume violations (TVV)) and PVA. Two subtypes of PVA, referred to here as double-trigger asynchrony (DTA) and breath stacking asynchrony (BSA), cause varying degrees of incomplete exhalation in between breaths resulting in a phenomenon referred to as dynamic hyperinflation^4,5,7^. DTA occurs when a ventilator’s set inspiratory time (I-time) is shorter than a patient’s desired “neural I-time”, with ongoing patient inspiratory effort at the termination of inspiratory support resulting in the immediate triggering of a second breath without intentional exhalation (Fig. 1a). DTA can result in substantially larger than intended TVi delivery despite otherwise optimal selection of ventilator settings^3,18,20,27^. BSA, like DTA, is characterized by incomplete exhalation in between breaths and is common in diseases with expiratory flow limitation such as acute exacerbations of asthma or chronic obstructive pulmonary disease (COPD)^7^. Unlike DTA, BSA results from either a ventilator-set or patient-triggered respiratory rate too fast to allow sufficient time for complete exhalation in between successive breaths (Fig. 1b and Supplementary Fig. S1). While both DTA and BSA result in dynamic hyperinflation, their distinct pathophysiologic mechanisms merit unique methods of detection. We thus developed and validated distinct rule-based classification algorithms to calculate inspiratory and expiratory TV (TVi and TVe, respectively), both DTA and BSA, and several common clinical artifacts (suctioning/auto-triggering of the ventilator, a subset of coughs, and transient disconnection from the ventilator) (Supplementary Table S2) that may result in the false-positive classification of artifacts as PVA. We aimed for TV accuracy within a pre-specified equivalence threshold of 10% relative to TV measured by the ventilator, and sensitivity and specificity of ≥90% for each PVA detection algorithm both before and after clinical artifact removal.

We hypothesized that the ventMAP engine would be able to measure TV with accuracy equivalent to a commercial ventilator, and that the recognition and algorithmic removal of clinical artifacts (referred to here as “artifact correction”) would significantly improve the specificity of PVA detection without compromising sensitivity. We present here the results of our derivation and validation studies.

## Results

**Figure 2:**
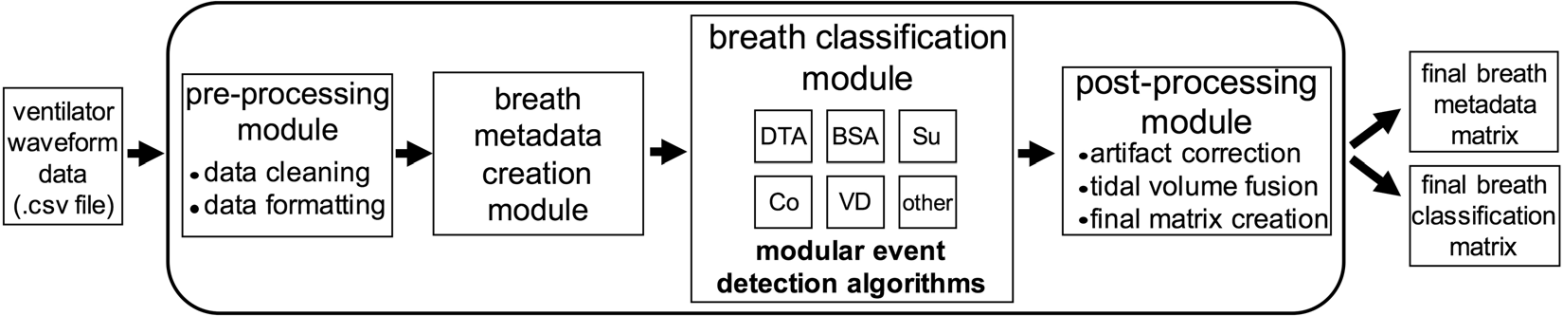
Schematic depiction of ventMAP’s modular architecture and standardized inputs and outputs.

To address existing limitations on waveform data access, we first developed an inexpensive open source architecture that allows continuous and unobtrusive collection of high-frequency pressure and flow waveform data from mechanical ventilators, and then developed an extensible, modular, multi-algorithm analytic software platform (ventMAP) grounded in the clinical rules used in bedside MV waveform analysis to automate the quantitative analysis of OTV (Fig. 2). We performed extensive pre-clinical simulation testing of individual component algorithms with further algorithm derivation and final validation using patient-derived data.

### Validation of Tidal Volume Measurement

Accurate measurement of TVi and TVe is required for the quantitative analysis of off-target TV, and provides essential breath-level metadata used for the algorithmic detection of both PVA and clinical artifacts (Fig. 1a–f). The Puritan Bennett model 840 (PB840) ventilators (Medtronic Corporation) used in our health system are accurate to within 10% of the set TV^34^, limited by the inherent imprecision of the ventilator’s flow sensor. We thus used a mechanical lung (QuickLung, IngMar Medical) to test the accuracy of ventMAP’s TVi and TVe measurement algorithms. Three separate experiments were performed using 3 different PB840s, testing ventMAP-derived TVi and TVe in a total of 1021 breaths across a range of ventilator modes, trigger mechanisms, set TV, and set inspiratory pressures using a pre-specified equivalence threshold of +/− 10%. ventMAP-derived TVi and TVe were equivalent to ventilator-derived TV across all measured conditions (Supplementary Table S1). The mean ventMAP-derived TVi and TVe, aggregated across all tested settings of both assist control-volume control and assist control-pressure control ventilator modes, were equivalent to the TVs recorded by the ventilator’s internal software (Table 1).

**Table 1:** Difference between ventMAP-calculated and ventilator-recorded tidal volumes in volume control and pressure control modes.

	TVi		TVe	
	% Difference	p-value	% Difference	p-value
AC/VC	3.1% [2.9–3.2]	p < 0.0001	5.0% [4.8–5.1]	p < 0.0001
AC/PC	5.1% [5.0–5.1]	p < 0.0001	5.0% [4.9–5.1]	p < 0.0001

Differences reported as mean difference, 95% confidence interval, and p-value for equivalence test with pre-specified equivalence margin of +/−10% (H_0_: Ventilator and ventMAP are not equivalent). Positive values indicate that ventilator volumes were larger than ventMAP volumes. AC/VC, assist control-volume control; AC/PC, assist control-pressure control; TVi, inspiratory tidal volume; TVe, expiratory tidal volume.

### Classification of Double-Trigger Asynchrony

Regions of interest containing a mix of DTA, BSA, and clinical artifacts from a derivation cohort with 5075 manually annotated breaths from 16 distinct patients were used for algorithm derivation. Clinical rules used for bedside DTA recognition were translated into a series of logical operations and encoded into the ventMAP rules engine (Fig. 1g,h and Supplementary Table S2), followed by iterative feature selection and parameterization to identify events in which a low ratio of TVe:TVi, indicating air trapping, was associated with a non-physiologic E-time and low exhaled volume (Supplementary Table S2). Algorithm performance was assessed for sensitivity, specificity, and overall accuracy using logistic regression to control for potential similarities in waveform characteristics within patients, and differential event rates between patients. Algorithm performance was compared to a gold standard classification data set derived from multi-clinician manual annotation of the same breaths.

In the derivation cohort, ventMAP achieved a sensitivity, specificity, and overall accuracy of 0.988, 0.965, and 0.967, respectively, for the classification of DTA. ventMAP performance was then tested without further modification in a separate validation data set consisting of 4644 manually annotated breaths from 17 mechanically ventilated patients. In the validation cohort, ventMAP’s performance decreased somewhat with sensitivity, specificity, and overall accuracy of 0.940, 0.920, and 0.922, respectively, but remained above our pre-specified goal of ≥90% for all three measures (Table 2).

**Table 2:** ventMAP performance metrics in the derivation and validation data sets.

Event Type	Derivation Data Set (n = 16)			Validation Data Set (n = 17)		
	Accuracy	Sensitivity	Specificity	Accuracy	Sensitivity	Specificity
Double Trigger	0.967	0.988	0.965	0.922	0.94	0.92
	[0.962, 0.971]	[0.972, 0.996]	[0.960, 0.970]	[0.914, 0.930]	[0.913, 0.960]	[0.912, 0.928]
Breath Stacking	0.984	0.985	0.984	0.977	0.967	0.98
	[0.980, 0.987]	[0.975, 0.992]	[0.980, 0.987]	[0.973,0.981]	[0.955, 0.977]	[0.975, 0.985]
Cough, Suction, Vent Disconnect Combined	0.992	0.907	0.995	0.981	0.879	0.989
	[0.989, 0.994]	[0.859, 0.943]	[0.993, 0.997]	[0.977, 0.985]	[0.841, 0.912]	[0.986, 0.992]

Data presented include means and [95% confidence limits].

### Classification of Breath Stacking Asynchrony

BSA classification rules were encoded to identify events in which a low ratio of TVe:TVi, indicating air trapping, was associated with a physiologic E-time (Fig. 1b and Supplementary Table S2 and Fig. S1). We defined the threshold value for the ratio of TVe:TVi as <90% because values ≥90% were too subtle for BSA identification by manual annotation and of questionable reliability given the ventilator’s imprecision of TV measurement (+/−10%)^34^. ventMAP achieved a sensitivity, specificity, and overall accuracy of 0.985, 0.984, and 0.984, respectively, in the derivation cohort. In the validation cohort, performance declined slightly with sensitivity, specificity, and overall accuracy of 0.967, 0.980, and 0.977, respectively (Table 2).

### Reduction in PVA Misclassification with Automated Clinical Artifact Correction

Our early experience with manual waveform annotation and algorithm development revealed that several VWD artifacts commonly observed during routine care shared morphologic similarities to PVAs of interest, resulting in false positive classification of PVA and TVV (Fig. 1d–f and Supplementary Figs S3–4). We thus developed algorithms to recognize several classes of clinical artifacts including patient suctioning, a subset of cough-related artifacts, and transient patient-ventilator disconnect events. The output of these algorithms was then used to drive a higher-order heuristic algorithm referred to as “artifact correction”, that transformed any detected PVA also recognized as a clinical artifact into the class “not PVA”. After optimization of artifact correction algorithms in our derivation cohort (Table 2), we tested ventMAP with and without the use of artifact correction in our validation cohort to test the hypothesis that artifact correction would improve the specificity of PVA detection without reducing sensitivity.

**Figure 3:**
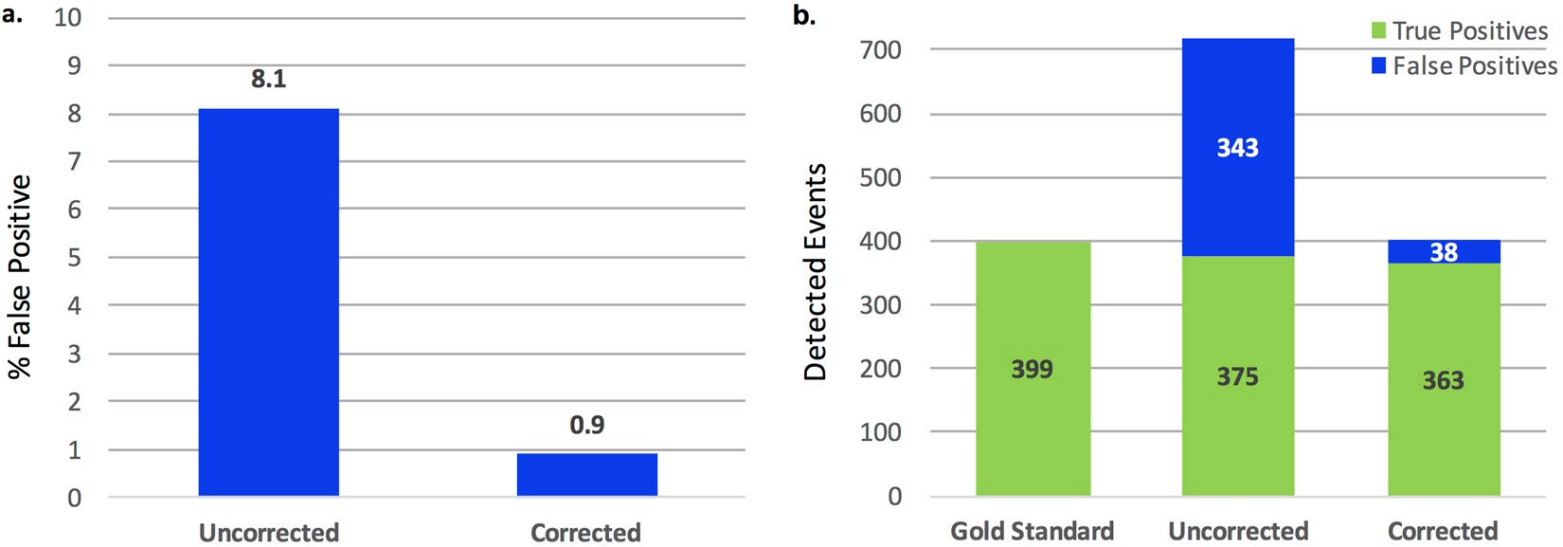
Artifact correction reduces false-positive event detection. (**a**) Change in double trigger false-positive detection rate with and without artifact correction in the validation data set (n = 4644 breaths). (**b**) Reduction in the number of detected double triggers in the validation data set with and without artifact detection.

In our derivation cohort, use of artifact correction resulted in a 2.8% [95% CI 0.9–4.7%; p = 0.006] improvement in DTA classification specificity and a non-significant 0.6% [95% CI −2.0–0.8%; p = 0.361] decrease in sensitivity (Table 3 and Supplementary Table S3) whereas artifact correction had no significant effect on BSA classification. In the validation cohort, we observed a 7.1% [1.1–13.2%; p = 0.024] improvement in the specificity of DTA classification and a non-significant 3.0% [−6.3–0.3%; p = 0.067] decrease in sensitivity. Artifact correction resulted in a 0.6% [0.2–1.0%; p = 0.009] improvement in the specificity of BSA classification, and a non-significant 0.3% [−0.9–0.2%; p = 0.189] decrease in sensitivity (Table 3 and Supplementary Table S3). Most notably, we observed a 9-fold reduction in the false-positive detection rate of DTA with the use of artifact correction resulting in a 44.2% decrease in the total number of detected DTA events from 718 without artifact correction to 401 breaths with use of artifact correction, with 399 true DTA events in the gold standard data set (Fig. 3 and Supplementary Table S4).

**Table 3:** Change in performance of PVA classification algorithms after the application of artifact correction.

	Derivation Data Set (n = 16)			Validation Data Set (n = 17)		
	Accuracy	Sensitivity	Specificity	Accuracy	Sensitivity	Specificity
Double Trigger	2.60%	−0.60%	2.80%	6.20%	−3.00%	7.10%
	[0.8, 4.3]	[−2.0, 0.8]	[0.9, 4.7]	[1.0, 11.4]	[−6.3, 0.3]	[1.1, 13.2]
	p = 0.007	p = 0.361	p = 0.006	p = 0.021	p = 0.067	p = 0.024
Breath Stacking	0.40%	−0.90%	0.60%	0.40%	−0.30%	0.60%
	[−0.1, 0.8]	[−1.7, 0.16]	[0, 12.7]	[0.03, 0.7]	[−0.9, 0.2]	[0.2, 1.0]
	p = 0.105	p = 0.021	p = 0.047	p = 0.036	p = 0.189	p = 0.009

Data are expressed as % change with [95% confidence intervals] and p-value from weighted least squares regression. Positive values indicate improved performance with artifact correction.

### Integration of Multi-Algorithm Output to Reclassify Tidal Volume Violations

DTA and BSA both lead to dynamic hyperinflation that can be quantified by calculating the sum of two successive TVis and subtracting the intervening TVe to yield a “fused” TVi that represents the effective distending volume for the lungs (Fig. 1h). Failure to account for the effective distending volume of a fused breath may lead to failure to detect associations between TVi and clinical outcomes in research studies, and may lead to volutrauma and worse clinical outcomes if clinically unrecognized and un-remedied^3,8,9,26^. Conversely, excessive detection of false positive OTV may bias research and lead to “alarm fatigue” if implemented in clinical decision support systems^35,36^. We thus developed a heuristic event classification algorithm referred to as “TV-fusion” that uses output from TV calculation, DTA classification, and artifact correction algorithms to fuse the component inspiratory and expiratory TVs of DTA breaths and output the effective distending TV of each DTA. We then calculated the mean TVi and the distribution of TVV (on-target versus off-target, and the relative severity of off-target breaths) across all DTA breaths in the validation cohort, with and without the use of the TV fusion algorithm.

**Figure 4:**
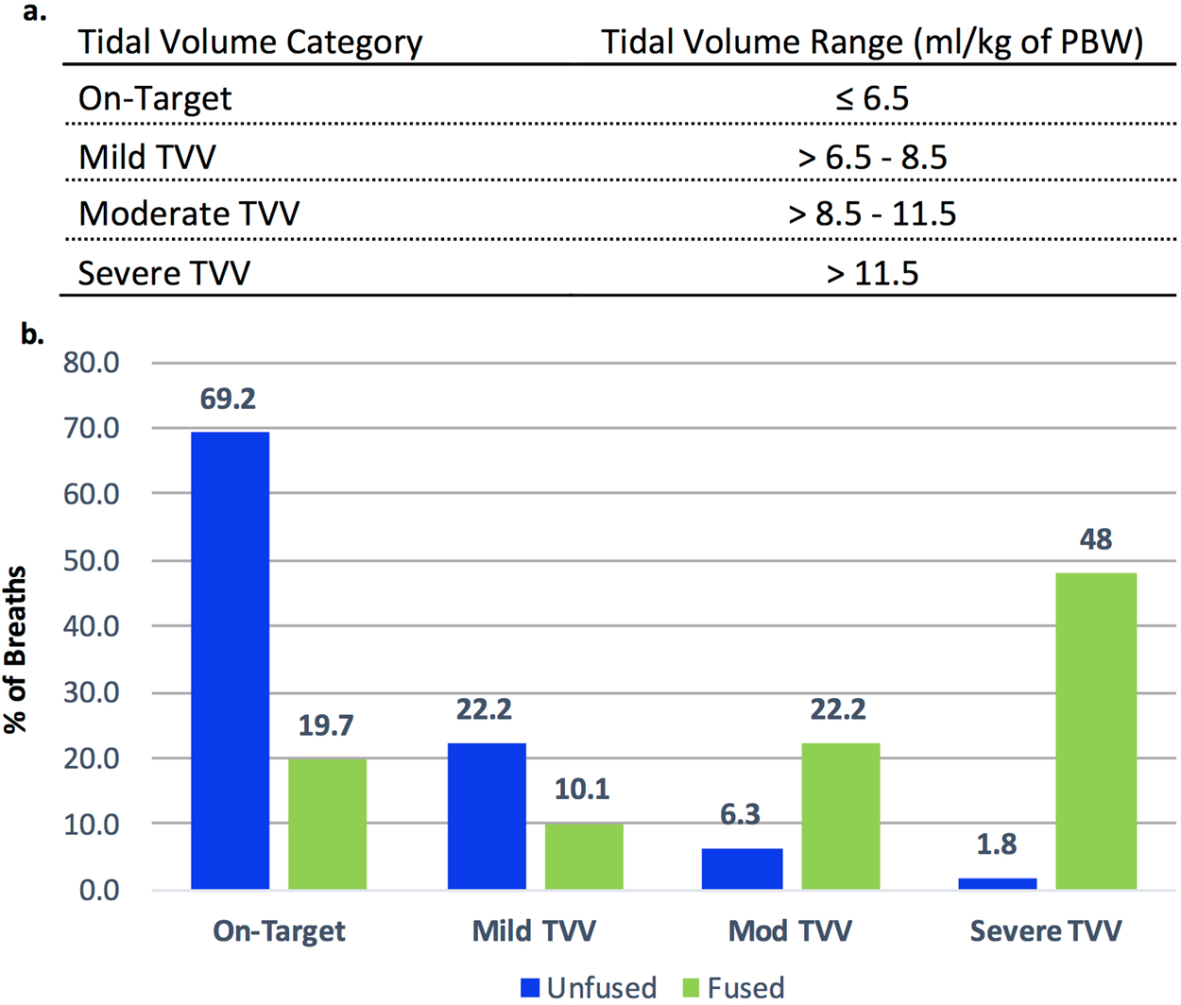
Tidal volume fusion changes the relative distribution of tidal volume violations. (**a**) Tidal volume violation ranges assessed by ventMAP for both fused and unfused breaths. (**b**) Change in the classification of tidal volume violations with the use of tidal volume fusion algorithm in double trigger asynchrony breaths from the validation cohort. Blue bars, double trigger component breaths without tidal volume fusion, green bars, with tidal volume fusion; TVV, tidal volume violations.

In the validation data set, mean TVi for DTAs was significantly higher when TV-fusion was employed, with a mean TVi of 293.3 ml (95% CI, 278.6–308.0) without TV-fusion and 562.2 ml (95% CI, 529.7–594.7; p < 0.0001 for the difference between means). Clinically, prescribed TV are based on predicted body weight (PBW) derived from sex and height, with TVi of ≤6.5 ml/kg of PBW representing the standard of care for patients with severe hypoxemic respiratory failure. We used the average height of a U.S. female to normalize all DTA TVs in the validation cohort before and after TV-fusion. After identifying all potential DTA breaths and removing false positives through artifact correction, we stratified off-target breaths with and without TV-fusion as mild, moderate, or severe based on the extent to which a given breath exceeded a target of ≤6.5 ml/kg (Fig. 4a). We found significantly more severe TVV amongst fused than unfused breaths (Fig. 4b), with a mean increase in TVV class of 1.58 [95% CI: 1.02–2.15, p < 0.0001] per breath.

## Discussion

In this study, we examined the accuracy of a novel, modular multi-algorithm analytic platform (ventMAP) to detect two main classes of off-target ventilation, namely TVV and PVA. We used customized Raspberry Pi microcomputers (Supplementary Fig. S5) to unobtrusively collect high frequency, high volume VWD, and tested ventMAP performance in a derivation-validation study design using clinician-annotated gold standard data including nearly 10,000 breaths from 33 patients including multiple ventilator modes and acute indications for MV. We showed that ventMAP can provide highly accurate breath-level quantitation of both TVi and TVe, highly sensitive and specific classification of two clinically relevant subtypes of PVA, and that removal of common clinical VWD artifacts can significantly improve the specificity of PVA detection without compromising sensitivity.

Our findings have several important implications. First, we have demonstrated the potential of inexpensive open source computer hardware and software solutions to overcome data access limitations to the use of high sampling rate streaming patient monitoring data. Historically, many devices in critical care environments are not interfaced with EHRs, or are interfaced to acquire infrequent data snapshots rather than the rich high volume, high velocity data streams typical of physiologic monitoring data. Previous studies that have acquired MV waveform data in electronic form have required physical connection through a laptop to each ventilator, placement of a monitoring device in-line in the MV circuit, or have used proprietary hardware and software limited to a small number of ICU beds^18–27^. These approaches may limit the amount of time and number of subjects that can be studied, may introduce observer bias^28,37^, or may be cost prohibitive in terms of hardware, software, and human resources required for data acquisition. In contrast, our data acquisition platform uses commercially available hardware costing approximately $75 US dollars per ventilator, occupies minimal space (Supplementary Fig. S5) allowing connection out of site on the back of each ventilator, and enables continuous wireless data acquisition and transmission over the enterprise network on multiple ventilators throughout the health system without need for human intervention after initial connection. At least one other group has developed a similar solution to data acquisition from patient monitoring devices using inexpensive open source computing resources^38^. These low-cost, open-source platforms demonstrate proof of concept that multidisciplinary research teams can overcome the technical and financial barriers to accessing patient-derived physiologic monitoring data for clinical and translational research in critical care, thereby democratizing a path to previously difficult to access data types.

Our findings also have important implications for the study of patient-ventilator interactions and the development of clinical decision support systems (CDSS) operating on waveform data derived from clinical devices. Several previous studies have applied automated analytic approaches to VWD. Approaches have included the use of commercial general purpose waveform analysis software^20,25^, power spectral analysis^23^, the development of custom rules-based algorithms^19,27^, and the development of proprietary software packages to analyze multiple subtypes of PVA^24,26^. While these studies have laid an important foundation for the automated analysis of patient-ventilator interactions, limitations remain including the need for detailed clinical validation of algorithm accuracy, the need for analytics that can detect multiple discrete types of PVA, and software that can detect relevant waveform artifacts to improve signal-to-noise detection. Our work extends these previous studies in several ways.

Using ventMAP’s modular design, we were able to validate separate OTV classification algorithms for the related PVA subtypes of DTA and BSA. Previous studies have not attempted to distinguish between these two PVA subtypes, either focusing on DTA at the expense of BSA^18–20,25,26^, or including DTA and BSA in one heuristic algorithm to detect dynamic hyperinflation^27^. While related, DTA and BSA have important mechanistic differences that we felt merited distinct classification strategies. In this regard, DTA has historically been defined as resulting from inspiratory effort persisting beyond the end of the ventilator’s programmed inspiratory time, resulting in the triggering of another supported breath with little or no exhalation in between breaths^4,5,18^. In contrast, we have taken a nuanced view, defining BSA empirically as incomplete exhalation in between breaths (TVe:TVi < 90%) with a longer period (>0.3 sec) of exhalation than observed typically in DTA, representing an attempted exhalation in between successive breaths (Fig. 1b and Supplementary Fig. S1). By enabling the distinct classification of DTA and BSA, we are able to capture breath stacking events across a wide spectrum of severity, detecting DTA events that may result in a doubling of intended TVi, as well as milder BSA events that may not meet previous definitions of breath stacking^18,26,27^ asynchrony, but may still result in substantial dynamic hyperinflation over time in patients with expiratory flow limitation such as those with asthma or COPD (Supplementary Fig. S1b)^4,5,7^. This “splitter’s” approach enables analysis of the associations between PVA subtypes and adverse outcomes across the entire spectrum of dynamic hyperinflation, allows event detection and decision support functionality specific to the distinct pathophysiologic mechanisms of each PVA subtype, and is key to the development of systems that will use detailed phenotyping and event detection to enable precision critical care management.

Whereas some previous studies have restricted event detection to dichotomous PVA classification, we developed ventMAP to enable sub-classification of OTV event severity using breath-level metadata derived from waveform processing such as inspiratory and expiratory times and volumes, minimum and maximum pressures or flows, etc (Supplementary Table S5). Graded event severity such as the net volume of trapped gas from a DTA or BSA event, or the degree of persistent end-expiratory flow in BSA, may allow parsing of the association between sub-classes of OTV and clinical outcomes, analogous to the use of gradations of the ratio of PaO2/FiO2 for prognostication and trial enrollment in the acute respiratory distress syndrome^6,9,39^. Graded OTV detection may allow the identification of important thresholds for the dose of OTV and/or OTV-subtypes required to precipitate lung injury, hemodynamic compromise, and patient discomfort. The ability to sub-classify OTV may be particularly important for the development of CDSS where the ability to refine alarm thresholds based on the type, frequency, and severity of OTV rather than its presence or absence alone may allow better matching of decision support to individual patient and provider needs. Future studies will need to define the optimal feature sets and parameter thresholds for event severity classification in relation to clinical outcomes to better define “clinically relevant” subtypes of OTV, a challenge relevant to the generation of knowledge from clinical big data sources in general.

Our work also highlights the potential benefits of artifact correction in the detection of OTV, and in the processing of complex patient-derived physiologic monitoring data more broadly. To our knowledge, no prior studies have attempted to identify common clinical artifacts in VWD to improve the specificity of OTV detection. Earlier studies of automated MV waveform analysis made no attempts to detect or correct for the presence of clinical artifacts, potentially limiting estimation of the prevalence of OTV^18–27^. Recent work by Beitler *et al*. supports the potential value of artifact correction where 4.7% of all recorded MV waveforms were manually removed from analysis during clinician review of waveform quality, including artifacts from endotracheal suctioning and ventilator disconnect^27^. Automated artifact correction has been studied elsewhere in critical care where correction of electrocardiogram chest compression artifact has been used to visualize the underlying cardiac rhythm to minimize breaks in chest compressions during cardiopulmonary resuscitation^40^. These efforts have led to at least one commercial product using this approach^41^, demonstrating the potential to translate such technologies to clinical application. Given the impracticality of manual artifact removal in large data sets or in real-time, and a relatively low OTV event rate across studies, automated artifact correction may significantly improve association studies that attempt to correlate OTV with important care processes or clinical outcomes, and may improve the acceptance and utility of CDSS designed to improve clinician responses to potentially dangerous or uncomfortable patient-ventilator interactions.

Finally, our work points to the potential value in the development of modular, extensible healthcare-specific analytic software where standardized outputs from individual analytic modules can be used combinatorially to recognize increasingly complex clinical events or states, such as with ventMAP’s TV-fusion functionality, while preserving computational efficiency and avoiding the introduction of unanticipated software dependencies. ventMAP’s modular architecture (Fig. 2) was designed to allow the development and testing of additional OTV detection algorithms over time at both the unit- and system-levels, including the potential incorporation of novel algorithms from other research groups, to test existing alternative event detection strategies, or validate the performance of novel algorithmic classification methods^42^. ventMAP’s development also underscores the value of working in multi-disciplinary teams of clinicians, informaticists, engineers, and computer scientists to address the informatics challenges facing the development and translation of healthcare big data analytics into clinical application including data access, transmission, storage, analysis, security, and information delivery^30–32^.

Our study has several strengths. First, our data acquisition infrastructure enables unobtrusive data collection to minimize observer effect^28,37^, and allows longitudinal and diverse sampling of patients, disease states, modes of MV, and selection of waveform regions of interest that represent a broad spectrum of both OTV and clinical artifacts. Our use of blinded multi-clinician waveform annotation to produce consensus gold standard data sets allowed for careful algorithm training and subsequent validation on a range of typical and atypical OTV waveform morphologies derived from real-world patient data. Similarly, our use of artifact correction enables a clearer picture of OTV event rates in heterogeneous waveform samples. Finally, our multi-disciplinary, team-based approach to the development of an extensible modular software platform allows uncomplicated incorporation of novel PVA and clinical artifact recognition algorithms to further extend ventMAP’s analytic capabilities.

Our study also has a number of limitations. First, our manual selection of ROIs for algorithm training may have introduced selection bias. We attempted to mitigate this issue through an extensive manual survey of patient waveforms during ROI selection for the derivation data set, and through semi-random ROI selection at very low magnification for the validation data set (Supplementary Fig. S6). Second, algorithm development included empiric feature selection and parameter thresholding using expert knowledge rather than using statistical or machine learning methods, which could have resulted in improved performance or more efficient algorithm derivation. Third, we did not compare ventMAP’s performance to previously published approaches^18,20,27^, since previous efforts have not attempted to distinguish between DTA and BSA, and have not attempted to correct for clinical artifacts. Nonetheless, it is possible that alternative methods would outperform ventMAP if compared directly and future work in the field will need to address alternative methods for the derivation of OTV detection algorithms, standardizing the definitions of common types of OTV and clinical artifacts, and the use of standards for data encoding to enable data sharing and the development of interoperable analytic software packages agnostic to ventilator type or data acquisition platform^43^. Fourth, ventMAP is able to detect some but not all of the clinical artifacts that may cause false-positive PVA classification. For example, waveform morphology in patients with an endotracheal tube cuff leak or bronchopleural fistula may be very similar to true BSA waveforms (in all cases TVe < TVi), such that ventMAP may overestimate BSA prevalence in patients with these conditions. Future research will need to model an increasingly diverse set of clinical artifacts, including the development of both morphologic and functional classification criteria such as when the detection frequency of a given artifact (e.g., BSA) exceeds physiologically plausible conditions (e.g., when the calculated amount of gas trapped in the chest from sequential BSA events exceeds predicted total lung capacity). Finally, our study included patients and clinician-annotators from a single health system, and future studies should include data from multiple institutions including both academic and community settings to optimize generalizability.

In summary, we have shown that a modular, multi-algorithm software platform (ventMAP) can automate the processing of high-volume VWD to detect common forms of OTV including TVV and PVA with high levels of sensitivity and specificity. We have also shown that common sources of clinical waveform artifacts can compromise the estimation of OTV frequency and that dedicated algorithms to detect and correct for the presence of artifacts can significantly improve the specificity of clinical event detection without compromising sensitivity. This work will help to enable future multi-center studies to delineate relationships between the cumulative dose and temporal distribution of OTV, and key patient-centered outcomes. Automated analysis of OTV will also facilitate the development of real-time clinical decision support systems to detect clinically relevant events not captured with existing methods (Supplementary Fig. S7). Future efforts to translate this and related research into scalable clinical decision support platforms will ultimately allow the effective use of computational systems in the delivery of precision critical care.

## Materials and Methods

### Data Acquisition

Studies of patient-ventilator interactions have been limited by difficulty in acquiring waveform data from mechanical ventilators, variously requiring proprietary software, additional instrumentation placed in-line in the ventilator circuit, and/or a direct laptop interface representing large barriers to data access in terms of human resources, cost, and potential bias introduced due to the observer effect^28,37^. To overcome these barriers, we developed a low cost, easy to deploy, open-source, and unobtrusive data acquisition platform that would allow for continuous VWD acquisition simultaneously from multiple patients anywhere in the hospital. We used Raspberry Pi (RPi) microcomputers running a version of the Linux operating system (Rasbian), secured out of sight on the back of Puritan Bennett model 840 (PB840) ventilators (Medtronic Corporation), and connected using a serial-USB null modem cable (StarTech, model ICUSB232FTN) attached to a serial port on the back of the PB840 screen. We developed custom scripts in the Python programming language to automatically connect to the health system enterprise network, acquire the current date and time, read the PB840’s serial port, and write ASCII-encoded pressure, flow, and time data at 50 Hz to disk. Discrete files were written every two hours to prevent data loss in the event of device failure, and to facilitate subsequent waveform visualization. Additional scripts were developed to automate hourly file back-ups to a networked study server, and an easy to use custom web-based application was developed to allow remote server-side file renaming with subject identifiers once data collection was complete, and to clear old data from RPis by study personnel with no technical background. The entire data acquisition platform and workflow was designed to be fully automated once an RPi was connected to the PB840 and plugged in.

### Algorithm Development and Validation

ventMAP was developed using the Python programming language and all described algorithms follow a rule-based framework in which logical rules for processing the data from each breath were derived from principles of bedside MV waveform interpretation^4,5,7^. In general, ventMAP processes arrays of pressure, flow, and time data from each recorded breath to derive a series of quantitative metadata such as TVi and TVe, inspiratory and expiratory times (I-time and E-time, respectively), peak inspiratory pressure, positive end-expiratory pressure (PEEP), and other derived variables (Supplementary Table S5). Derived metadata were used as features upon which logical rules were developed, with sets of related rules constituting an OTV or artifact event detection algorithm. Parameter thresholds for algorithm features were iteratively determined through trial and error using a gold standard derivation data set of known breath types as “ground truth”, and an initial classification matrix was populated showing all events detected for each breath. An additional algorithm was designed to process the initial classification matrix to recognize when a breath had been classified as both a clinical artifact and an OTV event, and modify the matrix to remove the OTV event. Similarly, another algorithm, referred to as TV-fusion, recognizes when a DTA event is present, and calculates the effective TVi by adding the two component TVis and subtracting the intervening small TVe if present (Fig. 1h).

TVi and TVe were calculated by integrating the area under the flow-time curve using Simpson’s method (fourth order accurate), from breath start until the first data point where flow has transitioned from positive (inspiratory) to negative (expiratory) (Fig. 1g). Experimental data were obtained by ventilating a calibration lung (Quicklung, Ingmar Medical) using PB840s in both assist control-volume control (AC/VC) and assist control-pressure control (AC/PC) modes, across a range of TVs and inspiratory pressures. Three different ventilators were used in separate experiments. Displayed TV were recorded manually from the ventilator user interface and compared to ventMAP TV estimates (n = 1021 breaths). ventMAP-derived TV estimates were tested for equivalence to PB840-recorded values by calculating the mean % difference between ventMAP and PB840 values, using a prespecified equivalence threshold of +/−10%, which was based on the +/−10% margin of error for TV calculation reported in the PB840’s technical manual^34^. T-tests were used for equivalence testing under each experimental condition (Supplementary Table S1). In addition to testing each experimental condition separately, we combined all VC mode experiments and all PC mode experiments and conducted equivalence testing for each mode. Estimates and standard errors were derived from a weighted least squares regression with weights for the number of breaths derived from each experiment. For the equivalence tests for the combined PC and VC modes, two one-sided t-tests were constructed from the point estimates and standard errors obtained from the weighted linear regression.

OTV event detection algorithms were developed for DTA, BSA, in-line suctioning, ventilator disconnect, and a subset of coughs (Fig. 1 and Supplementary Table S2). ventMAP algorithm performance was assessed for sensitivity, specificity, and overall accuracy compared to gold standard data sets generated from independent annotation of patient-derived waveforms by two Pulmonary and Critical Care physicians (JYA and BTK). Annotations were compared and disagreements were settled by re-review and consensus to arrive at a gold standard classification for each breath. A limited set of ventMAP-derived metadata including E-time and the ratio of TVe/TVi were available to reviewers during annotation to augment visual inspection but both reviewers were blinded to all ventMAP classification output. After gold standard generation, a derivation data set containing 5075 breaths from 16 unique patients was used for algorithm development, with 50–300 breath regions of interest selected to represent a broad spectrum of variation in OTV, artifacts, ventilator modes (AC/PC, PC/PC, AC/volume-targeted pressure control, and pressure support ventilation), and disease states (acute respiratory distress syndrome, acute asthma/COPD, and other). Algorithm development proceeded iteratively by logic refinement, parameter thresholding, and the incorporation of exception handling rules to account for intra-class variation in waveform morphology. Algorithm development proceeded until sensitivity, specificity, and accuracy were all >90% in the derivation data set. Once ventMAP code development stopped, final algorithm performance was assessed using a validation data set consisting of 4644 breaths from 17 unique patients. To minimize selection bias, breath regions of 50–300 breaths were selected for the validation data set by random file selection followed by waveform visualization at low magnification to identify regions of heterogeneity indicating the potential presence of OTV (Supplementary Fig. S6). Regions were examined at intermediate magnification and included in the validation data set if the OTV and/or artifact event rate appeared to be over 5%. All patient data were obtained as part of a study approved by the institutional review board (IRB) of the University of California at Davis. All methods were carried out in accordance with the guidelines and policies set forth by the IRB, and informed consent was obtained from all participants.

### Statistical Analysis

Analysis of algorithm performance in both the derivation and validation cohorts was conducted with and without correction for clinical artifacts. Each algorithm’s sensitivity, specificity, and overall accuracy were estimated using logistic regression. To account for varying numbers of breaths and events across patients (Supplementary Table S6) we used the patient-specific total number of breaths, number of asynchronous breaths, and number of non-asynchronous breaths present in the gold standard as weights in the logistic regression model when modeling overall accuracy, sensitivity and specificity.

Algorithm performance with and without artifact correction was compared by estimating the differences in sensitivity, specificity, and accuracy between the two results for each patient and then fitting a weighted least squares regression with weights as described above. The only term in the regression was the intercept term, which represents the difference in performance between the two classification sets. The model tested whether the intercept differed significantly from zero, accounting for the varying number of breaths and events across subjects. Weighted logistic regression was also used to compare differences in estimated TV with and without TV-fusion, and the difference in the proportions of TVV classes. For this analysis, TVV were classified as 0, 1, 2, and 3 for no violation (0) to severe violation (3), respectively (Fig. 4). To compare TVV violations, the ratings were considered integers. For each breath, the difference in ratings between fused and unfused estimates was calculated. These differences were then averaged for each patient in the validation cohort and a weighted linear regression with the number of breaths per patient as the weights was used to test for a difference between fused and unfused values. All results are presented as mean differences with 95% confidence intervals. All hypothesis tests were two-tailed and a pre-specified p-value of <0.05 for all comparisons was considered statistically significant. Statistical analyses were conducted using R Statistical Computing Language Version 3.3.1 (R Core Team 2016).

### Data availability

The datasets generated during and/or analyzed during the current study are available from the corresponding author on reasonable request.

## Electronic supplementary material

Supplementary Information
