# Supplementary material for: Development and Validation of a Multi-Algorithm Analytic Platform to Detect Off-Target Mechanical Ventilation
Source: Sci Rep. 2017 Nov 3;7:14980. doi: 10.1038/s41598-017-15052-x (PMC5670237; doi:10.1038/s41598-017-15052-x)
Supplement: Supplementary file 1 — Supplementary Information [file 41598_2017_15052_MOESM1_ESM.pdf]

Supplementary Information

Title: Development and Validation of a Multi-Algorithm Analytic Platform to Detect Off-Target Mechanical Ventilation

Authors: Jason Y. Adams[1*], Monica K. Lieng[2], Brooks T. Kuhn[1], Greg B. Rehm[3], Edward C. Guo[3], Sandra L. Taylor, [4], Jean-Pierre Delplanque[5], Nicholas R. Anderson[6]

Affiliations:
[1]Division of Pulmonary, Critical Care, and Sleep Medicine, University of California at Davis; Sacramento, CA.
[2]School of Medicine, University of California at Davis; Sacramento, CA.
[3]Department of Computer Science, University of California at Davis; Davis, CA.
[4]Department of Public Health Sciences, Division of Biostatistics, University of California at Davis; Davis, CA.
[5]Department of Mechanical and Aerospace Engineering, University of California at Davis; Davis, CA.
[6]Department of Public Health Sciences, Division of Informatics, University of California at Davis; Davis, CA.

*To whom correspondence should be addressed: Jason Y. Adams, MD MS, 4150 V St. Suite 3400, Sacramento, CA 95817, jyadams@ucdavis

Figure S1. Examples of additional breath stacking asynchrony morphologies.

Figure S2. Example of additional double trigger morphology observed in assist control-pressure control.

Figure S3. Example waveforms representing cough.

Figure S4. Examples of in-line suctioning morphology observed in different modes of mechanical ventilation.

Figure S5. Raspberry Pi microcomputer.

Figure S6. Workflow for selecting regions of interest (ROI) in the validation cohort.

Figure S7. Comparison between tidal volumes recorded in the electronic health record and by ventMAP.

Table S1. Percent difference between ventMAP calculated tidal volumes and tidal volumes recorded by Puritan Bennett model 840 ventilators.

Table S2. Dictionary of event classification algorithms currently available in the production instance of ventMAP.

Table S3. ventMAP performance metrics in the derivation and validation data sets after automated removal of clinical artifacts.

Table S4. Double trigger asynchrony event detection rates in the validation cohort with and without use of artifact correction algorithm.

Table S5 Dictionary of quantitative metadata describing each breath currently available in the production instance of ventMAP.

Table S6. Number and percentage of each event type over all patients in the derivation and validation data sets.

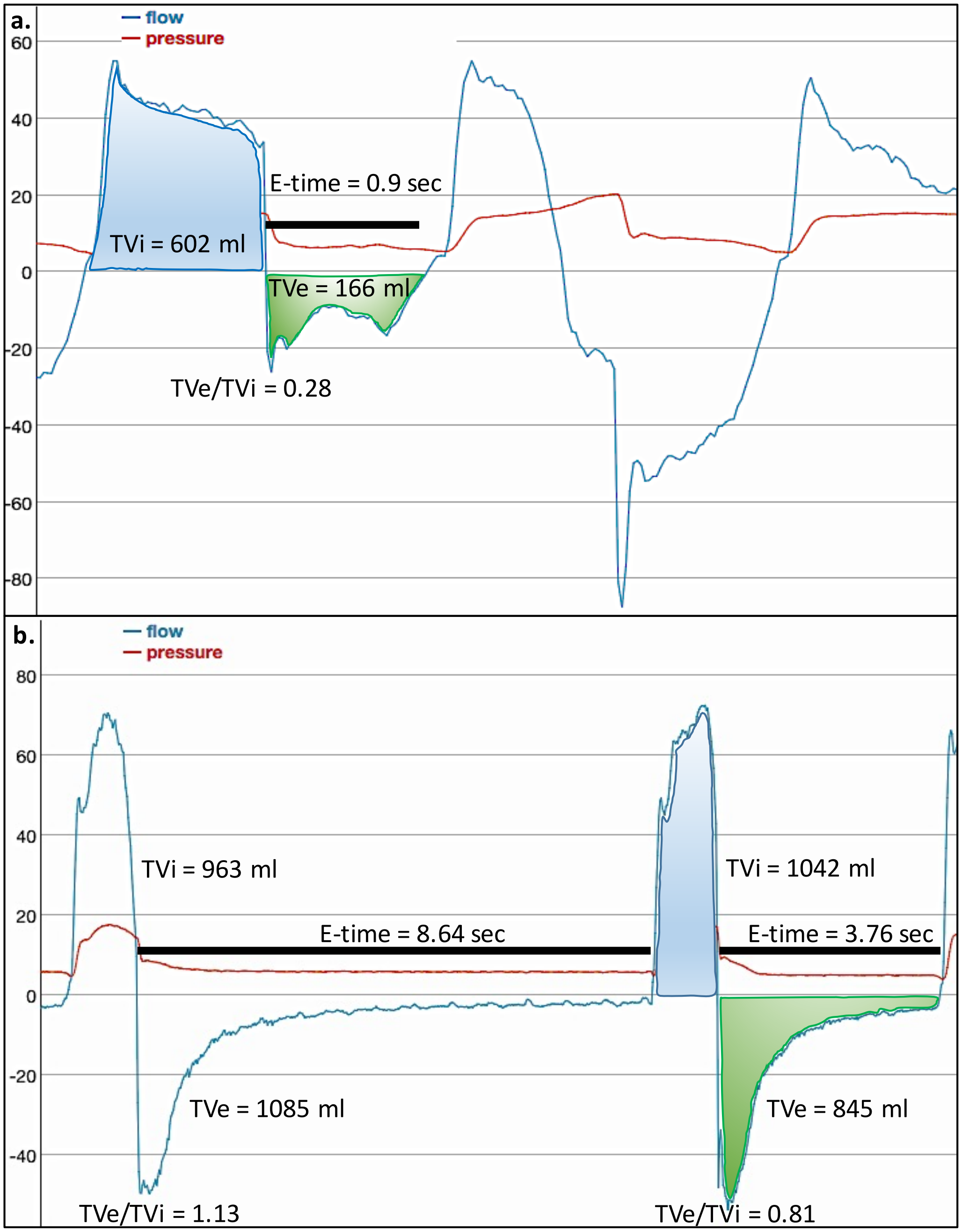

Figure S1. Examples of additional breath stacking asynchrony morphologies. a. Note the presence of a physiologic expiratory time and trapping of a large amount of inspired gas despite the absence of significant expiratory airflow limitation. **b.** Note the presence of substantial expiratory airflow limitation. Despite airflow obstruction, the first breath results in a net loss of gas from the thorax due to the long E-time, while the second breath's shorter E-time results in breath stacking. TVi, inspiratory tidal volume; TVe, expiratory tidal volume; E-time, expiratory time.

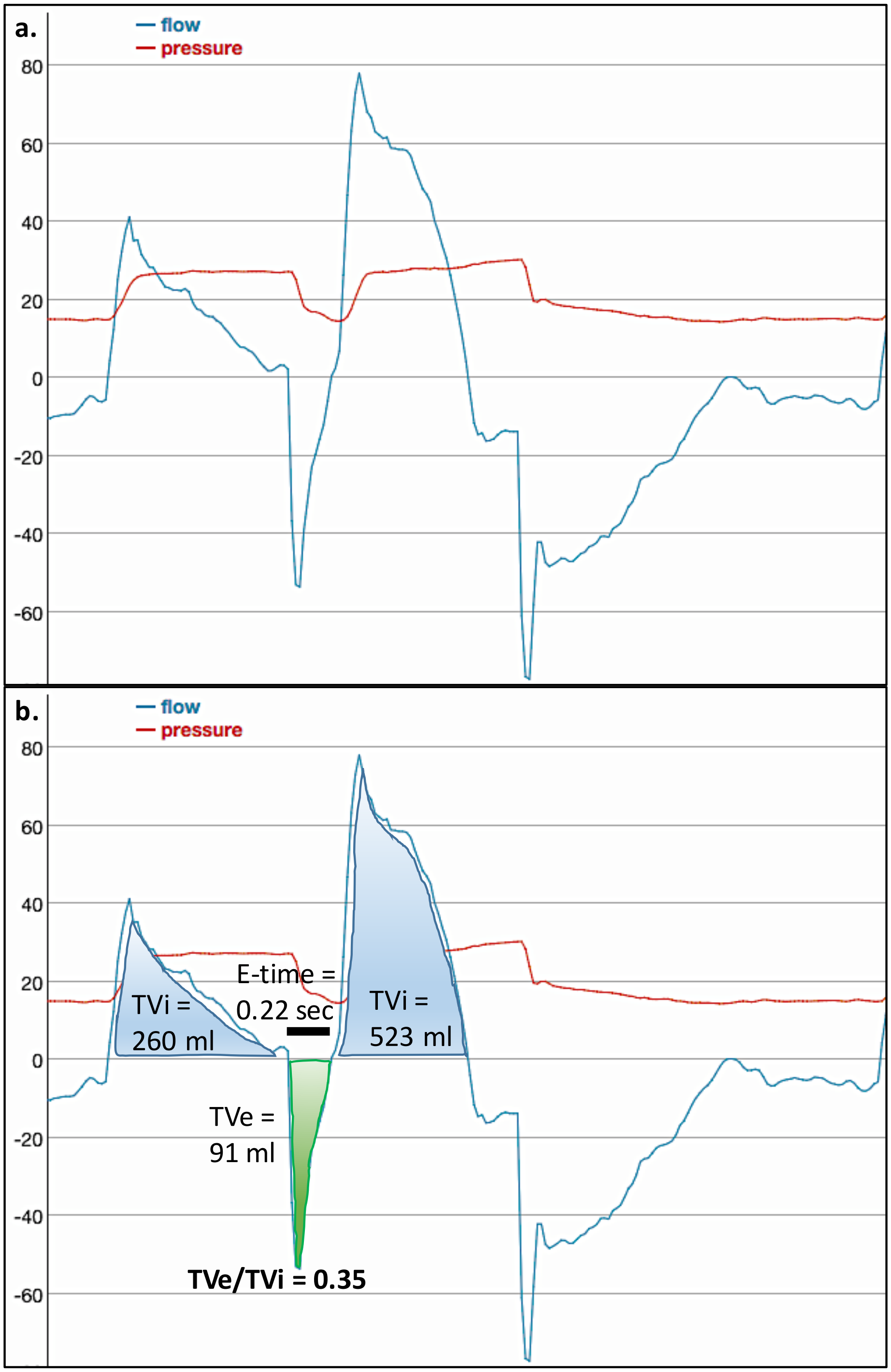

Figure S2. Example of additional double trigger morphology observed in assist control-pressure control mode where the first TVi is relatively small leading to a higher ratio of TVe/TVi than typically seen in a canonical double trigger breath in either AC/PC or AC/VC. Note the preservation of a non-physiologic expiratory time and associated small TVe < 100 ml. TVi, inspiratory tidal volume; TVe, expiratory tidal volume; E-time, expiratory time.

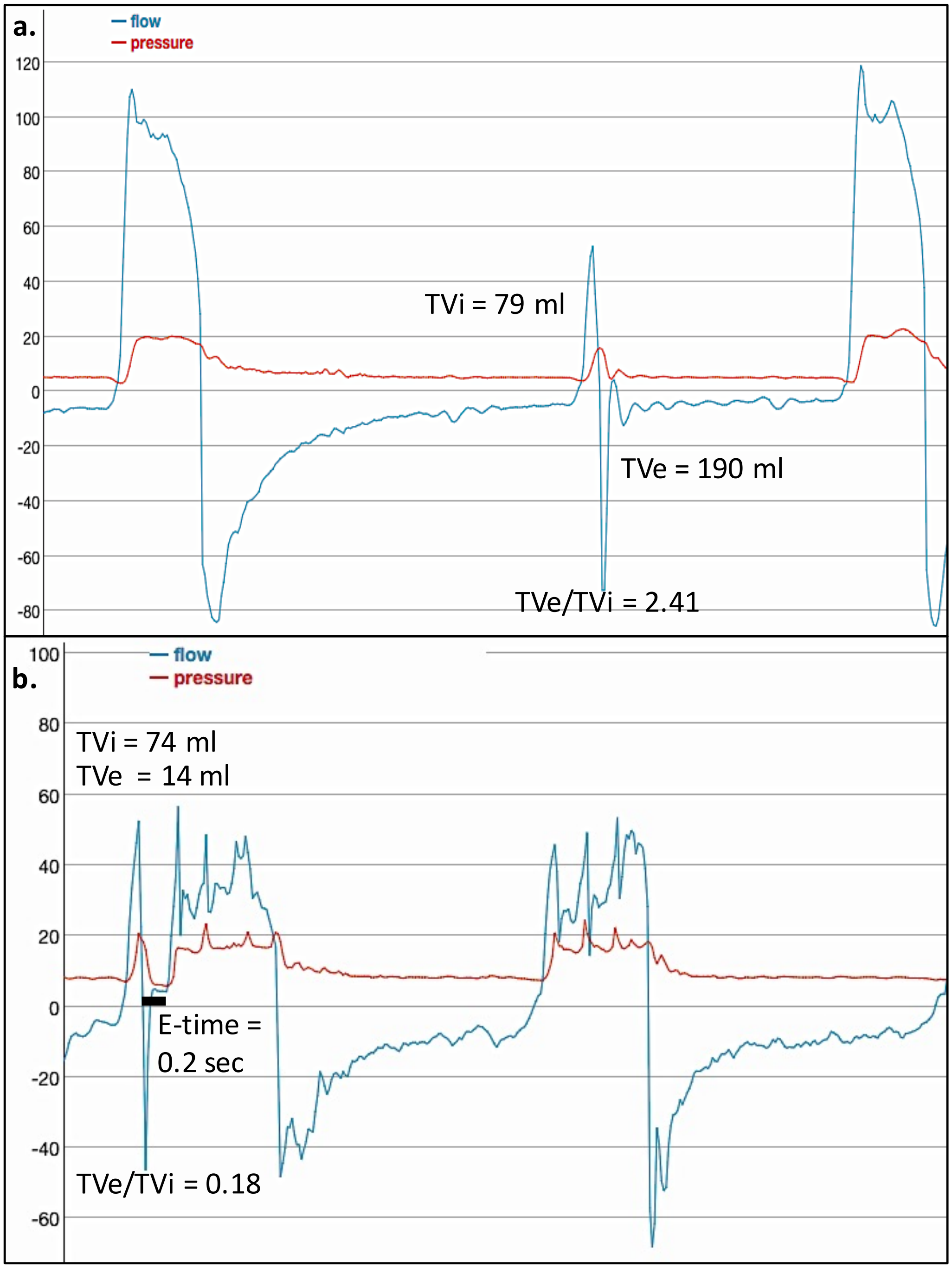

Figure S3. Example waveforms representing cough. A. Isolated cough in between two normal breaths. **B.** Cough followed immediately by two normal breaths. Note that this cough, followed immediately by a normal breath, would be classified as a double trigger asynchrony without use of artifact correction. Also note the presence of irregularities in both the flow- and pressure-time curves indicative of secretions in the endotracheal tube and/or circuit.

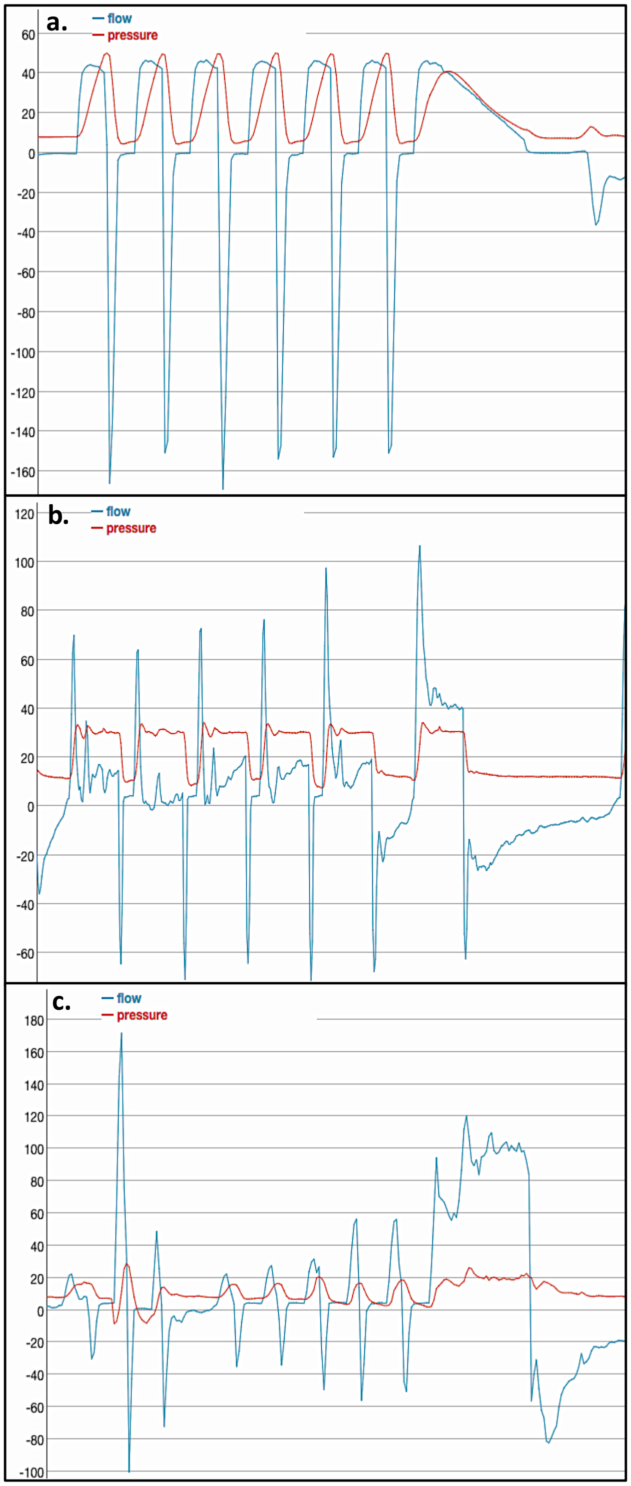

Figure S4. Examples of in-line suctioning morphology observed in different modes of mechanical ventilation. A. Suctioning in AC/VC **B.** Suctioning in AC/PC **C.** Suctioning in PS. Note the repeating instances of breaths with non-physiologic expiratory times resulting from suction-related auto-triggering. AC/VC, assist control-volume control mode; AC/PC, assist control-pressure control mode; PS, pressure support mode.

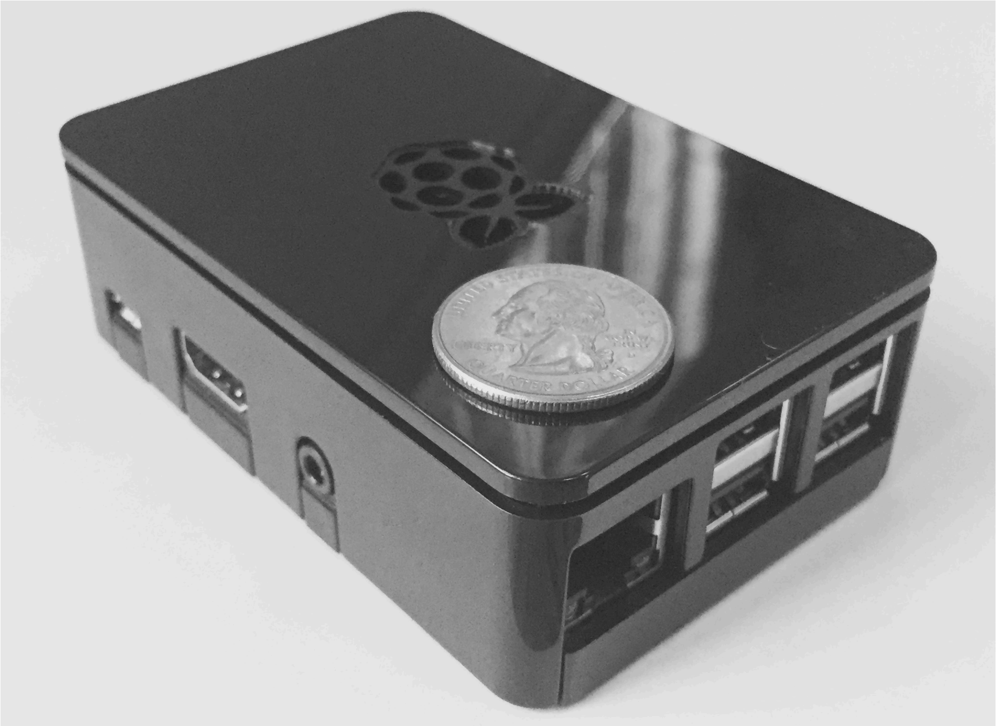

Figure S5. Raspberry Pi microcomputer used to continuously acquire and transmit ventilator waveform data over the health system wireless network to a study server. Raspberry Pis are connected to a serial port on the back side of the ventilator user interface screen using an RS232-to-USB null modem and secured out of sight.

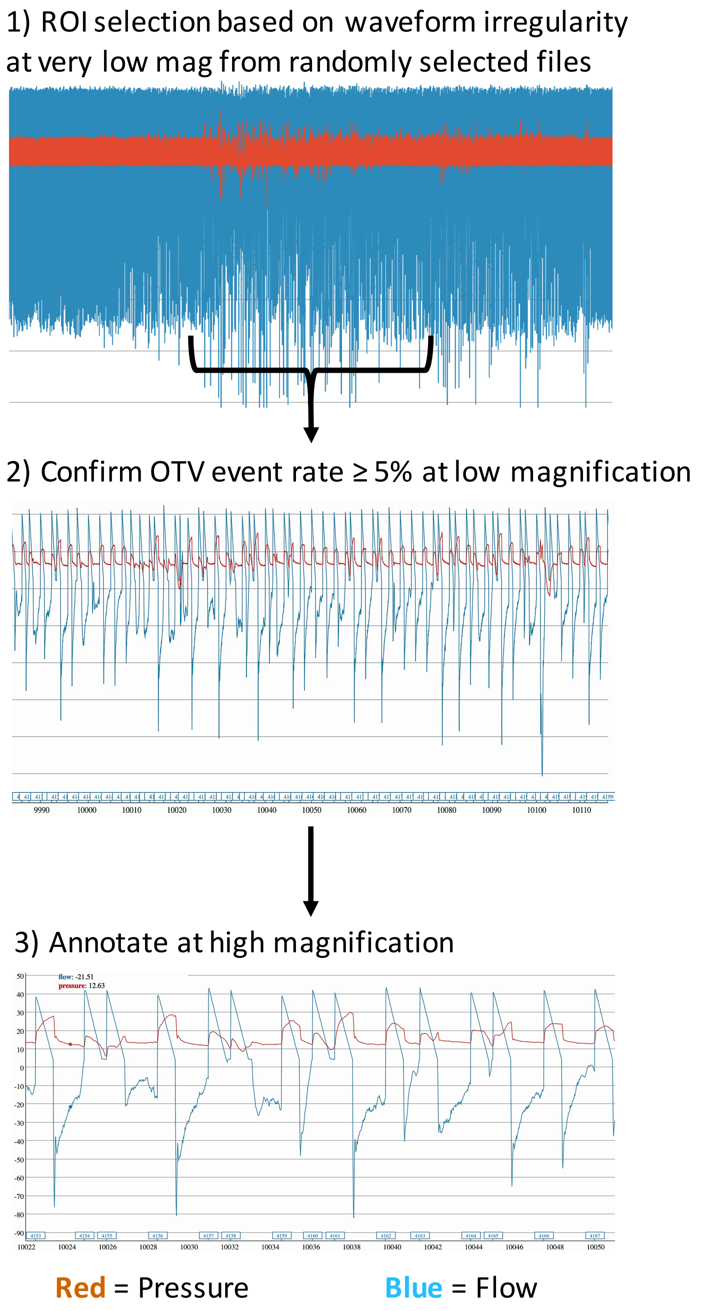

Figure S6. Workflow for selecting regions of interest (ROI) in the validation data set, minimizing bias with use of random file selection and very low magnification ROI selection.

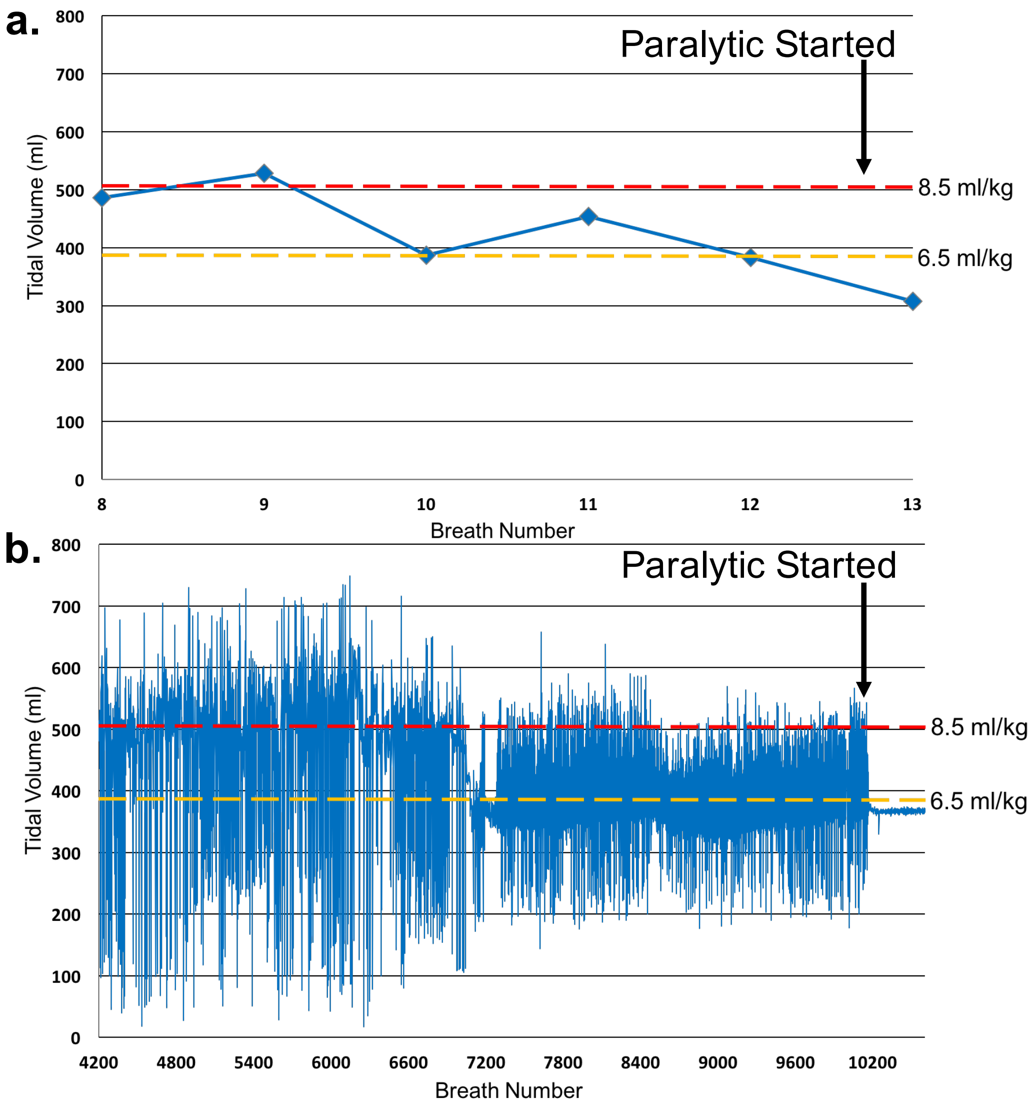

Figure S7. Comparison between tidal volumes recorded in the EHR and by ventMAP. A. Inspiratory tidal volumes recorded in the electronic health record in the 4 hours prior to the start of pharmacologic muscle paralysis in a patient with severe ARDS. **B.** Inspiratory tidal volumes, including fused double trigger asynchronies, recorded by ventMAP in the same 4-hour period prior to paralysis. Yellow dashed line indicates the upper limit of "on-target" tidal volumes for this patient. Red dashed line indicates the upper limit of "mild" tidal volume violations.

Ventilator Setting	N	TVi			TVe		
		Mean	LCL	UCL	Mean	LCL	UCL
PC5	37	0.07	0.068	0.072	0.052	0.05	0.054
PC10	42	0.041	0.04	0.041	0.041	0.04	0.042
PC15	41	0.057	0.052	0.063	0.039	0.038	0.041
VC250	37	0.039	0.035	0.042	0.058	0.054	0.062
VC500	40	0.053	0.051	0.054	0.094	0.082	0.106
VC750	38	0.057	0.057	0.058	0.069	0.061	0.076
PC300_Vtrig	103	0.05	0.048	0.052	0.045	0.044	0.046
PC600_Vtrig	112	0.05	0.05	0.051	0.057	0.056	0.057
VC1200_Vtrig	109	0.025	0.024	0.025	0.043	0.043	0.044
VC300_Vtrig	107	0.003	0.002	0.004	0.066	0.065	0.066
VC450_Vtrig	135	0.017	0.016	0.017	0.042	0.041	0.042
VC600_Ptrig	104	0.053	0.053	0.054	0.077	0.077	0.078
VC600_Vtrig	116	0.054	0.054	0.055	0.022	0.02	0.023
All PC	335	0.051	0.05	0.051	0.05	0.049	0.051
All VC	686	0.031	0.029	0.032	0.05	0.048	0.051

Table S1. **Percent difference between ventMAP calculated tidal volumes and tidal volumes recorded by Puritan Bennett model 840 ventilators** in volume control and pressure control modes. Differences reported as mean and 95% confidence interval; p-values were < 0.0001 for all equivalence tests with pre-specified margin of +/- 10% (H_0: Ventilator and ventMAP are not equivalent). Positive values indicate that ventilator volumes were larger than ventMAP volumes. TVi: inspiratory tidal volume; TVe: expiratory tidal volume; N, number of breaths recorded for each condition; LCL: lower confidence limit; UCL: upper confidence limit; PC: assist control-pressure control; PC"x" indicates the amount of applied pressure in centimeters of H_2O; VC: assist control-volume control; VC"x" indicates the set inspiratory tidal volume in milliliters; VTrig: flow trigger; PTrig: pressure trigger.

Event Name	Classification Matrix Name	Lay Description	Pseudocode
On-target TV	TVV_0	Any inspiratory TV ≤ 6.5 ml/kg of PBW	IF TVi or TVi_fused ≤ 6.5 ml/kg of PBW = TVV_0
Mild TVV	TVV_1	Any inspiratory TV > 6.5 - 8.5 ml/kg of PBW	IF 6.5 ml/kg of PBW < TVi or TVi_fused ≤ 8.5 ml/kg of PBW = TVV_1
Moderate TVV	TVV_2	Any inspiratory TV > 8.5 - 11.5 ml/kg of PBW	IF 8.5 ml/kg of PBW < TVi or TVi_fused ≤ 11.5 ml/kg of PBW = TVV_2
Severe TVV	TVV_3	Any inspiratory TV > 11.5 ml/kg of PBW	IF TVi or TVi_fused > 11.5 ml/kg of PBW = TVV_3
Double trigger asynchrony	DTA	2 successive breaths with a non-physiologic E-time and a low ratio of TVe/TVi	[E-time ≤ 0.3 sec AND TVe/TVi < 0.25], OR [E-time ≤ 0.3 sec AND TVe/TVi = 0.25-0.5 AND TVe < 125 ml] AND ≠ "any artifact" = DTA
Breath stacking asynchrony	BSA	2 successive breaths with a physiologic E-time and a ratio of TVe/TVi < 0.9	[E-time > 0.3 sec AND TVe/TVi < 0.9 sec] AND ≠ "any artifact" = BSA
Mild BSA	BSA_1	2 successive breaths with a physiologic E-time and a ratio of TVe/TVi between 0.66 and 0.89	[E-time > 0.3 sec AND 0.65 < TVe/TVi < 0.9 sec] AND ≠ "any artifact" = BSA
Moderate BSA	BSA_2	2 successive breaths with a physiologic E-time and a ratio of TVe/TVi between 0.33 and 0.65	[E-time > 0.3 sec AND 0.32 < TVe/TVi < 0.66 sec] AND ≠ "any artifact" = BSA
Severe BSA	BSA_3	2 successive breaths with a physiologic E-time and a ratio of TVe/TVi < 0.33	[E-time > 0.3 sec AND TVe/TVi < 0.33 sec] AND ≠ "any artifact" = BSA

Suction	Su	3 or more successive breaths with a non-physiologic E-time; adds the last breath in a suction cluster even if that breath has a physiologic E-time to account for common variability in the last auto-triggered breath	if current breath E-time ≤ 0.3 sec, then add 1 to suction counter (label breath as su1); if next breath E-time ≤ 0.3 sec, AND the previous breath E-time ≤ 0.3 sec, then add 1 to suction counter (label breath as su2); if next breath E-time ≤ 0.3 sec AND previous breath E-time ≤ 0.3 sec, then add 1 to suction counter (label breath as su3); if current breath E-time > 0.3 sec AND previous frame = E-time ≤ 0.3 sec, then add 1 to the suction counter (label breath as su4); if current breath E-time > 0.3 sec AND previous frame E-time > 0.3 sec, then counter is reset and current breath remains 0; final breath class = "suction" IF suction counter total count > su2 AND initial breath class = su"n" where n = any number of contiguous suction counts from 1..n
Cough	Co	identifies a subset of coughs with DTA- or BSA-like breath features that may result in false-positive PVA classification	if I-time ≤ 0.2 and ipAUC < 5 AND peak inspiratory flow > 20 L/min
Ventilator Disconnect	VD	identifies auto-triggering that occurs upon patient disconnect from the ventilator that, only in AC/PC mode, results in maximum flow delivery and high apparent inspiratory tidal volumes	TVi > 2000 ml AND ipAUC > 5

Table S2. **Dictionary of event classification algorithms** currently available in production instance of ventMAP.

	Derivation Data Set			Validation Data Set		
Event Type	Accuracy	Sensitivity	Specificity	Accuracy	Sensitivity	Specificity
**Double Trigger**	0.993 [0.990, 0.995]	0.982 [0.964, 0.993]	0.993 [0.991, 0.995]	0.984 [0.981, 0.988]	0.909 [0.878, 0.935]	0.992 [0.988, 0.994]
**Breath Stacking**	0.988 [0.984, 0.990]	0.975 [0.963, 0.984]	0.99 [0.987, 0.993]	0.981 [0.977, 0.985]	0.964 [0.951, 0.974]	0.986 [0.982, 0.990]

Table S3. **ventMAP performance metrics** (mean and 95% confidence limits) in the derivation and validation data sets after automated removal of clinical artifacts (cough, suction, and ventilator disconnects).

	Artifact Correction	
**Event Detected**	**Uncorrected**	**Corrected**
Total DTA Events	718	401
True Positive DTA	375	363
False Positive DTA	343	38
Gold Standard DTA	399	399
Gold Standard non-DTA	4245	4245

Table S4. Double trigger asynchrony event detection rates in the validation cohort (n = 4644 breaths) **with and without use of artifact correction** algorithm. The number of double trigger and non-double trigger breaths in the gold standard data set are also listed. DTA, double trigger asynchrony.

Variable Name	Units	Description
ventBN	integer	breath number as defined by the PB840 in a MV waveform file; note that values may repeat over time
BN	integer	relative breath number assigned to each breath in a MV waveform file
Absolute BS	datetime	absolute date and time of breath start, defined by the PB840 "BS" marker, in a MV waveform file
Absolute BE	datetime	absolute date and time of breath end, defined by the PB840 "BE" marker, in a MV waveform file
Relative BS	seconds	relative time assigned to breath start, defined by the PB840 "BS" marker, in a MV waveform file
Relative BE	seconds	relative time assigned to breath end, defined by the PB840 "BE" marker, in a MV waveform file
Absolute x0	datetime	absolute date and time of the first point after flow crosses consistently from positive (inspiratory) to negative (expiratory) flow
Relative x0	datetime	relative date and time assigned to the first point after flow crosses consistently from positive (inspiratory) to negative (expiratory) flow
TVi	milliliters	inspiratory tidal volume, defined as the integral of the flow-time curve values from BS to [x0 minus 1 time point]
TVe	milliliters	expiratory tidal volume, defined as the integral of the flow-time curve values from x0 to BE
TVe/TVi	unitless	the ratio of expiratory tidal volume to inspiratory tidal volume
TVi_fused	milliliters	when double trigger asynchrony (DTA) classification criteria are met, the effective TVi is calculated by adding the first TVi and second TVi and subtracting the first TVe to yield TVi_fused. The second TVe is counted as TVe_fused, and one breath is subtracted from the total breath count in the file to reflect the fusion event when counting the DTA event rate.
TVe_fused	milliliters	when DTA criteria are met, the second TVe in the two DTA component breaths is counted as the fused TVe without any mathematical manipulation
I-time	seconds	the time from BS to [x0 minus 1 time point]
E-time	seconds	the time from x0 to BE
I:E ratio	unitless	the ratio of the I-time to the E-time
BT	seconds	total breath time from BS to BE
RR	unitless	instantaneous respiratory rate, defined as the [60/BT]
PIF	liters/minute	peak inspiratory flow, defined as the maximum positive flow recorded from BS to [x0 minus 1 time point]
PEF	liters/minute	peak expiratory flow, defined as the most negative flow recorded from x0 to BE

PIP	cm H_2O	peak inspiratory pressure, defined as the maximum recorded pressure from BS to [x0 minus 1 time point]
minIP	cm H_2O	minimum inspiratory pressure, defined as the lowest recorded pressure from [BS plus 5 time points] to [x0 minus 1 time point]; [BS plus 5 time points] was used to define the start of the analytic window to exclude low pressures during the normal early rise in inspiratory pressure during a breath
ipAUC	cm H_2O	the inspiratory pressure area under the curve, defined as the integral of the pressure-time curve from BS to [x0 minus 1 time point]
epAUC	cm H_2O	the expiratory pressure area under the curve, defined as the integral of the pressure-time curve from x0 to BE
PEEP	cm H_2O	positive end-expiratory pressure, defined as the average of the last 5 data points from the pressure-time curve of each breath
PEF-BE	unitless	the slope of the line defined by the points PEF and BE on the flow-time curve for each breath
[PEF+0.16]-BE	unitless	the slope of the line defined by the points [0.16 seconds after PEF] and BE on the pressure-time curve for each breath
Dynamic compliance	cm H_2O	defined as TVi/[PIP-PEEP] for each breath
Paw	cm H_2O	mean airway pressure

Table S5. **Dictionary of quantitative metadata describing each breath** currently available in production instance of ventMAP software.

	Derivation		Validation	
Event Type	Number	Percentage	Number	Percentage
**Double Trigger**	334	6.6	399	8.5
**Breath Stacking**	849	16.7	1096	23.6
**Cough**	63	1.2	63	1.3
**Suction**	114	2.2	257	5.5
**Ventilator Disconnect**	5	0.09	5	0.1
**Cough, Suction, Vent Disconnect Combined**	182	3.5	323	6.9

Table S6. Number and percentage of each event type over all patients in the derivation and validation data sets.
